# Social connectedness and digital literacy in later life: the mediating roles of perceived barriers and social integration

**DOI:** 10.3389/fpubh.2026.1849056

**Published:** 2026-09-09

**Authors:** Ke Jiang, Yang Song

**Affiliations:** 1 School of Advertising and Branding, Communication University of China, Beijing, China; 2 The Wharton School, University of Pennsylvania, Philadelphia, PA, United States

**Keywords:** digital literacy, mediation, older adults, perceived barriers, social connectedness, social integration

## Abstract

**Background:**

Population aging and digital transformation are two major global trends; however, the potential pathways through which social connectedness may be associated with digital literacy in later life remain underexplored. This study examined the mediating roles of perceived barriers and social integration in the relationship between social connectedness and digital literacy among Chinese older adults.

**Methods:**

A cross-sectional survey was conducted among 505 middle-aged and older educated Chinese adults aged 50–70 years. Digital literacy was assessed across five dimensions. Social connectedness was measured using the Lubben Social Network Scale-6, perceived barriers using an 8-item scale adapted from the Technology Acceptance Model, and social integration using a Chinese version of the social participation scale. Logistic regression and mediation analyses were performed using bootstrap resampling, with demographic covariates controlled.

**Results:**

Regression results showed that social connectedness was significantly associated with digital literacy (*β* = 0.22, *p* < 0.001). Mediation analyses revealed that both perceived barriers (indirect effect: 0.0305, 95% CI [0.0120–0.0520]) and social integration (indirect effect: 0.1265, 95% CI [0.0940–0.1600]) served as significant mediators, with social integration accounting for a substantially larger proportion of the total effect (60.6% vs. 14.6%).

**Conclusion:**

These findings suggest that social connectedness is associated with digital literacy through two separate mediating pathways: higher levels of perceived barriers and lower levels of social integration. Interventions may wish to consider addressing both technological anxiety and social network strengthening, particularly for older adults with lower social connectedness.

## 1 Introduction

Population aging and digital transformation are two major global trends occurring in parallel. In China, the population aged 60 years and above has surpassed 300 million, accounting for more than 21% of the total population. At the same time, the number of older internet users has grown rapidly, making them one of the fastest-growing segments of the online population. Digital literacy, broadly defined as the ability to access, evaluate, create, and communicate information using digital technologies in a safe and effective manner, has become an essential skill for navigating this increasingly digitalized society. Digital literacy is a multidimensional construct that encompasses both functional skills (e.g., information retrieval, content creation) and socially embedded competencies (e.g., communication, critical evaluation). However, research indicates that digital literacy among older adults in China remains suboptimal. A cross-sectional study (1) of 2,086 older adults found that while most were internet users, their digital skills were predominantly limited to basic information acquisition, and only a minority effectively used digital tools for life management or health purposes. Moreover, compared to younger age groups, older adults have significantly lower rates of internet usage and lower eHealth literacy scores (2). Insufficient digital literacy not only limits older adults’ ability to access information and online services but may also exacerbate their social marginalization and relative deprivation (3). For example, digital literacy among Chinese older adults is differentially associated with subjective health, with entertainment-oriented digital use showing no beneficial effects (1), while life management information and smart healthcare device use positively contributed to well-being. Furthermore, low levels of education, income, and poor health status have been identified as key factors (4) constraining digital literacy among rural older adults in China, suggesting that disadvantaged groups face compounded barriers to digital inclusion.

Social connectedness (SC) refers to the strength and breadth of an individual’s social network, encompassing both family and friend relationships, and has been increasingly recognized as a significant public health concern in later life (5). A growing body of evidence indicates that lower social connectedness is associated with adverse health outcomes among older adults, including cognitive decline, reduced psychological well-being, and elevated risks of depression and loneliness (5). In recent years, researchers have begun to explore the relationship between social connectedness and digital technology use. On one hand, digital technologies—such as videoconferencing, social media, and instant messaging—have been widely promoted as tools to mitigate social isolation by facilitating older adults’ social connectedness with family and friends (6). On the other hand, digital exclusion itself has been conceptualized as a phenomenon that predisposes older individuals to social exclusion (7), suggesting a bidirectional relationship between the two. However, existing studies have predominantly focused on how digital literacy alleviates social isolation, whereas the reverse question—how lower social connectedness may be associated with lower digital literacy—has received considerably less empirical attention (6). This unidirectional perspective overlooks a critical reality: older adults who lack social connections often lack both the motivation to learn digital skills and the social support essential for acquiring them. Therefore, considering social connectedness as a key factor associated with digital literacy, rather than merely an outcome, is necessary for a more comprehensive understanding of the digital divide in later life.

The Technology Acceptance Model (TAM) posits that perceived ease of use is a core factor influencing technology adoption (8). For older adults, perceived barriers (PB) include age-related physical limitations, cognitive difficulties, technophobia, distrust, and lack of assistance. These factors represent major obstacles to digital engagement (9). From this perspective, perceived barriers serve as an internal psychological filter. Lower social connectedness may be related to perceived barriers in two ways. First, older adults with lower social connectedness lack technical guidance and support from family, friends, or community, making them more likely to view digital operations as “too difficult” or “impossible to learn” (5). Second, lower social connectedness may be associated with lower self-efficacy, intensifying anxiety and avoidance toward new technologies. Thus, perceived barriers constitute one mediating pathway through which lower social connectedness may be associated with lower digital literacy. Meanwhile, active aging theory emphasizes that social participation is a key pathway for older adults to maintain vitality and enhance well-being (10). Social integration (SI) refers to the degree to which individuals engage in community activities, interact with neighbors, and establish social connections. Unlike perceived barriers, social integration represents an external social resource. It provides opportunities for observational learning, peer support, and technological demonstration, serving as important social capital for digital literacy development (11). Older adults with lower social connectedness, with smaller social networks and lower interaction frequency, struggle to access information, help, and encouragement related to digital skills (5). Hence, social integration represents a second mediating pathway, conceptually distinct from perceived barriers. Empirical evidence confirms that perceived barriers are negatively associated with digital literacy, while social integration is positively associated. In this study, we test these two mediating pathways separately rather than in a serial mediation model. Drawing on Social Cognitive Theory (SCT) (12, 13), we conceptualize these pathways as operating through two conceptually distinct mechanisms. First, perceived barriers (PB) represent internal personal factors—including technological anxiety, low self-efficacy, and perceived difficulty—that may directly impede digital engagement. Second, social integration (SI) represents external environmental factors—such as social support, observational learning, and community engagement—that may facilitate digital literacy development. These two mechanisms—internal psychological barriers and external social resources—are theoretically distinct. While SCT suggests that personal and environmental factors interact, we focus on testing their independent mediating roles separately, without positing a specific sequential order between them. Methodologically, testing two separate simple mediation models allows us to examine the unique indirect effect of each mediator while maintaining theoretical clarity and avoiding unnecessary assumptions about the order of influence between the two mechanisms.

Despite these theoretical arguments, empirical evidence testing this dual-pathway model remains scarce. Although individual associations have been reported, few studies have directly tested the dual mediation model that positions social connectedness as an antecedent of digital literacy. This gap has limited the development of interventions that go beyond technical training to address the psychosocial roots of digital exclusion.

To address these research gaps, we conducted a cross-sectional study among 505 Chinese adults aged 50–70 years. By measuring social connectedness, perceived barriers, social integration, and digital literacy using validated scales, this study aims to: (1) examine the association between social connectedness and digital literacy; (2) test the mediating role of perceived barriers in the relationship between social connectedness and digital literacy; and (3) test the mediating role of social integration in this relationship. The findings will clarify the mechanisms underlying the digital divide in later life and provide empirical evidence for designing targeted interventions that address both technological anxiety and social network strengthening, particularly for older adults with lower social connectedness.

## 2 Materials and methods

### 2.1 Study population

This study focused on community-dwelling middle-aged and older adults in China. Based on literature indicating that digital literacy challenges emerge around age 50 and become more pronounced after 70, the target population was defined as individuals aged 50 to 70 years. Participant recruitment was conducted between August and October 2022 using convenience sampling through online platforms and community channels. A total of 716 questionnaires were returned. After rigorous data cleaning, 505 valid questionnaires were ultimately obtained, yielding an effective response rate of 70.5%. Inclusion criteria were: (1) age 50 to 70 years; (2) ability to complete the questionnaire either independently or with assistance from family members or interviewers; and (3) voluntary participation. Exclusion criteria were: (1) presence of severe cognitive impairment or mental illness; (2) inability to cooperate in completing the questionnaire. This study was approved by the Academic Committee of the School of Advertising and Branding, Communication University of China (approval No. XSLL20251107-6). All respondents provided informed consent prior to participation. The study ensured anonymity and confidentiality, and respondents retained the right to withdraw from the study at any time.

### 2.2 Demographic characteristics

Demographic information was collected using a self-administered questionnaire. Age was categorized into two groups for analysis: 51–60 years and 61–70 years. Gender (male = 1, female = 0) and retirement status (retired = 1, not retired = 0) were dichotomized. Educational level was measured on an ordinal scale and subsequently collapsed into three categories for analysis: junior high school or below, high school or technical school, and associate degree or above. Monthly income was also collapsed into three categories: low income (≤4,999 RMB), middle income (5000–9,999 RMB), and high income (≥10,000 RMB). Grandchildren status was categorized as none, one, or two or more. Household size was categorized as 1–2 persons, 3 persons, or 4 persons or more.

### 2.3 Questionnaire survey

Given the specific characteristics of the middle-aged and older adult population (e.g., visual impairment, unfamiliarity with technical terminology), this study employed a mixed-mode assessment approach combining self-administered scales and assisted evaluation. Questionnaires were distributed through an online platform and community channels. Respondents were permitted to complete the questionnaire independently or with assistance from family members or interviewers, this mixed-mode design was intended to include older adults with varying levels of digital literacy and comprehension, thereby reducing potential selection bias. The questionnaire consisted of three sections, including several validated scales and self-developed scales.

#### 2.3.1 Digital literacy scale

Digital literacy was measured using a scale developed based on the EU DigComp 2.0 and UNESCO Digital Literacy Global Framework (DLGF), with localized adaptations for the Chinese context. An initial item pool covering five domains (operational skills, information, communication, content creation, and security awareness) was constructed from existing scales and refined through expert meetings and focus group interviews with six older adults. Exploratory factor analysis (EFA) reconstructed digital literacy into five core dimensions comprising 47 items. The Information Acquisition and Application (IAA) dimension (16 items) measured information retrieval, storage, sharing, and digital life applications (Cronbach’s *α* = 0.848). The Basic Knowledge and Norms (BKN) dimension (14 items) assessed fundamental operations, online ethics, and security awareness (Cronbach’s *α* = 0.764). The Digital Content Creation (DCC) dimension (9 items) measured audio/video editing, content re-creation, and copyright awareness (Cronbach’s *α* = 0.887). The Digital Interaction and Communication (DIC) dimension (5 items) assessed online expression, opinion sharing, and topic participation (Cronbach’s *α* = 0.890). The Digital Information Evaluation (DIE) dimension (3 items) measured information credibility judgment and critical thinking (Cronbach’s *α* = 0.755). All items were rated on a 5-point Likert scale (1 = strongly disagree, 5 = strongly agree). The overall digital literacy score (DL) was calculated as the mean of the five dimension scores. The Cronbach’s α for the total scale was 0.906, indicating good reliability.

To further validate the five-factor structure of the digital literacy scale, we conducted a confirmatory factor analysis (CFA) using the MLR (maximum likelihood estimation with robust standard errors) estimator. The model demonstrated acceptable to good fit: *χ*^2^/df = 1.66, CFI = 0.952, TLI = 0.947, RMSEA = 0.036 (90% CI [0.032, 0.041]), SRMR = 0.042. All standardized factor loadings were statistically significant (*p* < 0.001), ranging from 0.46 to 0.81. Detailed CFA results are presented in Supplementary Table S3.

#### 2.3.2 Social connectedness, perceived barriers, and social integration

Social connectedness (SC) was measured using the 6-item Lubben Social Network Scale (LSNS-6), which assesses family and friend networks across three dimensions: frequency of contact, ability to confide, and availability of assistance. Items were rated on a 6-point scale (0 = none, 5 = nine or more), with higher scores indicating stronger social networks (i.e., greater social connectedness). The Cronbach’s α for this scale was 0.809. Perceived barriers (PB) were measured using an 8-item scale adapted from the perceived ease of use dimension of the Technology Acceptance Model. The scale assesses obstacles to digital participation, including age-related physical limitations, cognitive difficulties, technophobia, distrust, and lack of assistance (e.g., “too difficult to learn,” “no one to teach me”). Items were rated on a 5-point Likert scale (1 = strongly disagree, 5 = strongly agree), with higher scores indicating greater perceived barriers. An 8-item perceived usefulness scale was also tested but showed poor validity in exploratory factor analysis and was therefore excluded from the final model. The Cronbach’s α was 0.838. Social integration (SI) was assessed using an 8-item Chinese version of the social participation scale, measuring initiative in neighborhood interaction and community engagement (e.g., “I actively visit neighbors I have not seen for days,” “I can help older adults neighbors with shopping or chores”). Items were rated on a 5-point Likert scale (1 = strongly disagree, 5 = strongly agree), with higher scores indicating greater social integration. The Cronbach’s α for this scale was 0.819.

### 2.4 Quality control

All research assistants involved in data collection and cleaning received standardized training on survey procedures and quality control protocols prior to the study. To ensure data quality, multiple quality control measures were implemented. During the questionnaire design phase, a pilot study and focus group interviews (involving six eligible older adults) were conducted to optimize item wording, and an attention-check question (“Have you ever landed on the moon?”) was embedded to identify random responses. During data collection, channel-specific monitoring was employed by comparing completion rates (number of completions/number of visits) to identify anomalous channels, and response time distributions were analyzed to exclude suspected bot-generated responses concentrated within specific time periods. During data cleaning, questionnaires with completion time less than 360 s were excluded based on pre-test benchmarks, those answering “yes” to the attention-check question were removed, and bot-generated responses were identified and excluded through open-ended answer comparisons.

### 2.5 Data analysis

Statistical analyses were performed using R software (version 4.2.1). Descriptive statistics were calculated for all variables. Normality of continuous variables was assessed and confirmed prior to analysis; normally distributed continuous variables are presented as means and standard deviations (*M* ± SD). Differences in digital literacy across demographic subgroups were examined using independent *t*-tests (for two groups) or one-way ANOVA (for three or more groups). Additionally, percentiles (min, p5, p10, p25, median, p75, p90, p95, max) were calculated to describe the distributional characteristics of the core variables. Pearson correlation coefficients were computed to examine bivariate associations among the main study variables. One-way ANOVA was used to compare digital literacy across tertile groups of social connectedness (SC), perceived barriers (PB), and social integration (SI).

Linear regression models were fitted to examine the association between social connectedness and digital literacy. Five models were constructed: Model 1 was unadjusted; Model 2 adjusted for age and gender; Model 3 additionally adjusted for education and retirement status; Model 4 further adjusted for income, grandchildren, and household size; Model 5 added response mode, perceived barriers (PB) and social integration (SI) as mediators. Mediation analyses were performed using the mediation package in R. We tested separate simple mediation models for perceived barriers and social integration. Drawing on Social Cognitive Theory, perceived barriers are conceptualized as internal personal factors and social integration as external environmental factors. These two mechanisms are theoretically distinct and operate in parallel. Testing separate models allows us to examine each indirect effect independently without imposing a sequential order between the two mediators. Simple mediation models were tested separately for PB and SI to examine their indirect effects in the relationship between SC and DL. Bias-corrected bootstrap methods with 5,000 resamples were used to generate 95% confidence intervals (CIs) for indirect effects; effects were considered statistically significant if the CIs did not include zero. All regression models controlled for demographic covariates including age, gender, education, income, retirement status, grandchildren, and household size, response mode. Statistical significance was set at *p* < 0.05 (two-tailed).

## 3 Results

### 3.1 Descriptive statistics

The study sample consisted of 505 Chinese adults aged 50–70 years. As shown in Table 1, significant differences in digital literacy were observed across demographic subgroups. Females reported higher digital literacy than males (3.87 ± 0.52 vs. 3.75 ± 0.57, *p* = 0.011). Participants aged 51–60 years had higher digital literacy than those aged 61–70 years (3.84 ± 0.50 vs. 3.65 ± 0.75, *p* = 0.036). Digital literacy increased with educational level (*F* = 37.15, *p* < 0.001) and income level (*F* = 18.02, *p* < 0.001), with those holding associate degrees or above and those with high incomes reporting the highest scores. Retired participants had lower digital literacy than those not retired (3.72 ± 0.61 vs. 3.88 ± 0.49, *p* = 0.003). Notably, participants with two or more grandchildren had significantly lower digital literacy than those with none (3.73 ± 0.65 vs. 3.87 ± 0.47, *p* = 0.01).

**Table 1:** Digital literacy differences by demographic factors and tertile groups of social connectedness, perceived barriers, and social integration.

Variable	Category	*n*	DL (Mean ± SD)	Statistic	*p*-value
Gender	Male	236	3.75 ± 0.57	*t* = −2.57	0.011
	Female	269	3.87 ± 0.52		
Age	51–60 years	431	3.84 ± 0.50	*t* = 2.13	0.036
	61–70 years	74	3.65 ± 0.75		
Education	Junior high school or below	50	3.52 ± 0.63	*F* = 37.15	<0.001
	High school/technical school	135	3.68 ± 0.63		
	Associate degree or above	320	3.92 ± 0.46		
Insurance	Low income (≤4,999 RMB)	197	3.70 ± 0.59	*F* = 18.02	<0.001
	Middle income (5000–9,999 RMB)	220	3.85 ± 0.49		
	High income (≥10,000 RMB)	88	3.98 ± 0.51		
Retirement	Not retired	306	3.88 ± 0.49	*t* = 3.00	0.003
	Retired	199	3.72 ± 0.61		
Grandchildren	None	245	3.87 ± 0.47	*F* = 6.61	0.01
	One	125	3.80 ± 0.55		
	Two or more	135	3.73 ± 0.65		
Household size	1–2 persons	197	3.80 ± 0.53	*F* = 0.87	0.351
	3 persons	138	3.93 ± 0.47		
	4 persons or more	170	3.74 ± 0.61		
Response mode	Self-administered	458	3.90 ± 0.55	*t* = 5.77	<0.01
	Assisted	47	3.30 ± 0.70		
SC_group	High SC	135	4.06 ± 0.46	*F* = 25.00	<0.001
	Moderate SC	200	3.80 ± 0.52		
	Low SC	170	3.64 ± 0.56		
PB_group	High PB	147	3.59 ± 0.61	*F* = 44.53	<0.001
	Moderate PB	184	3.73 ± 0.50		
	Low PB	174	4.10 ± 0.40		
SI_group	High SI	156	4.14 ± 0.41	*F* = 71.75	<0.001
	Moderate SI	177	3.84 ± 0.44		
	Low SI	172	3.50 ± 0.57		

**Figure 1:**
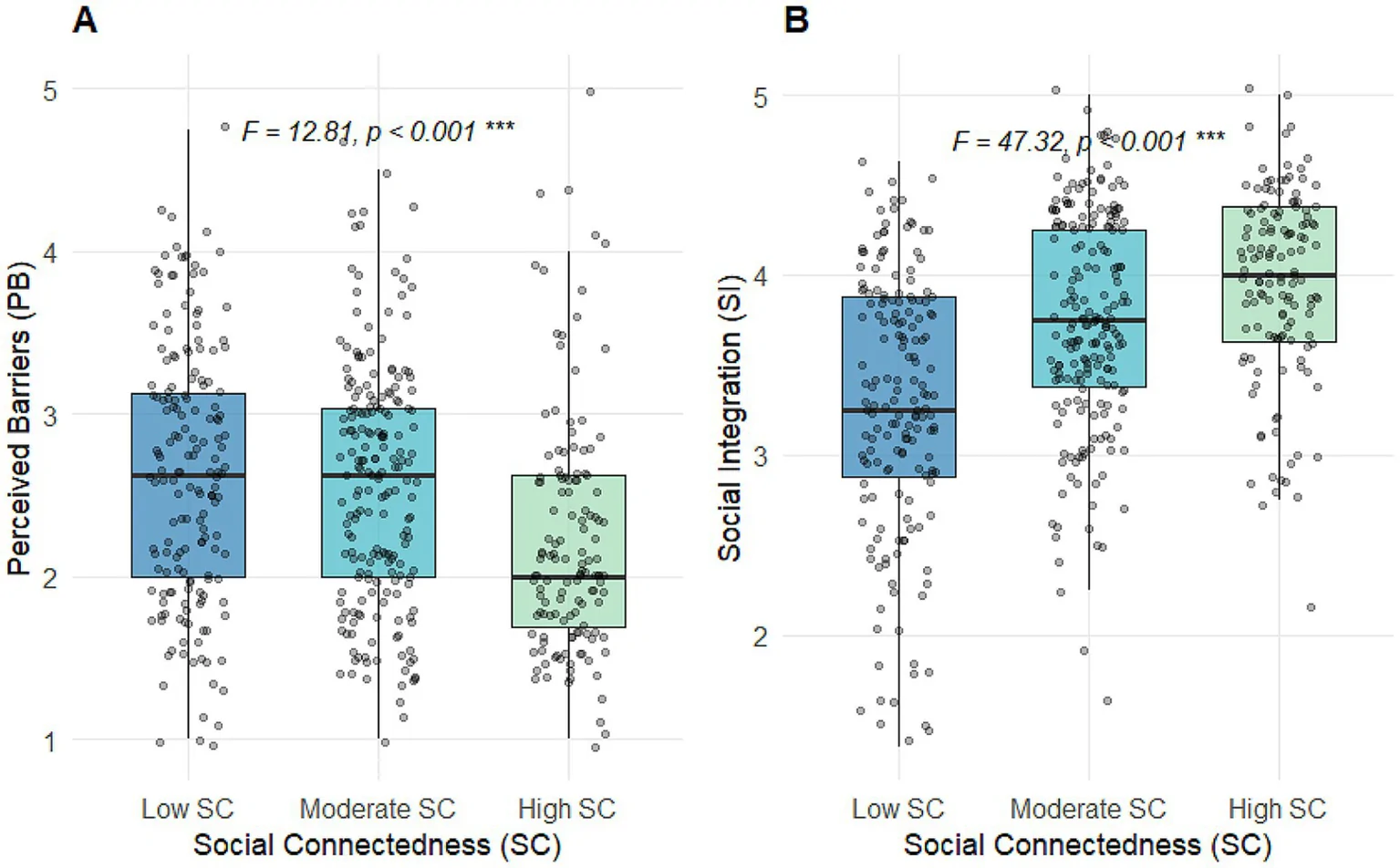
Perceived barriers and social integration across different levels of social connectedness. SC_group was created by dividing each variable into tertiles (low, moderate, high). Higher SC scores indicate stronger social networks. All comparisons were performed using one-way ANOVA. **(A)** Perceived barriers (PB) by social connectedness (SC) group. **(B)** Social integration (SI) by social connectedness (SC) group.

SC, social connectedness; PB, perceived barriers; SI, social integration; DL, digital literacy; SC_group, PB_group, and SI_group were created by dividing each variable into tertiles (low, moderate, high). The tertile groups (low, moderate, high) were created only for descriptive purposes (Table 1 and Figure 1) to visually illustrate patterns and for one-way ANOVA comparisons. These groupings were not used in the inferential models.

When categorized by tertiles, digital literacy increased progressively from low to high SC groups (*F* = 25.00, *p* < 0.001), and from high to low PB groups (*F* = 44.53, *p* < 0.001), and from low to high SI groups (*F* = 71.75, *p* < 0.001). These patterns suggest that higher social integration and lower perceived barriers are associated with better digital literacy.

### 3.2 Bivariate associations and group comparisons

Pearson correlation analysis (Supplementary Figure S1) revealed that all five digital literacy dimensions (BKN, IAA, DIC, DIE, DCC) were positively and significantly intercorrelated (*r* = 0.24–0.63). Figure 1 presents the distributions of perceived barriers (PB) and social integration (SI) across the three levels of social connectedness (Low, Moderate, High SC). One-way ANOVA revealed a significant effect of SC group on PB (*F* = 12.81, *p* < 0.001). Similarly, SC group had a significant effect on SI (*F* = 47.32, *p* < 0.001). SI increased monotonically with higher SC, with the High SC group showing the greatest social integration and the Low SC group the lowest. These patterns suggest that stronger social networks (higher SC) are associated with lower perceived barriers and greater social integration.

### 3.3 Regression analysis

**Figure 2:**
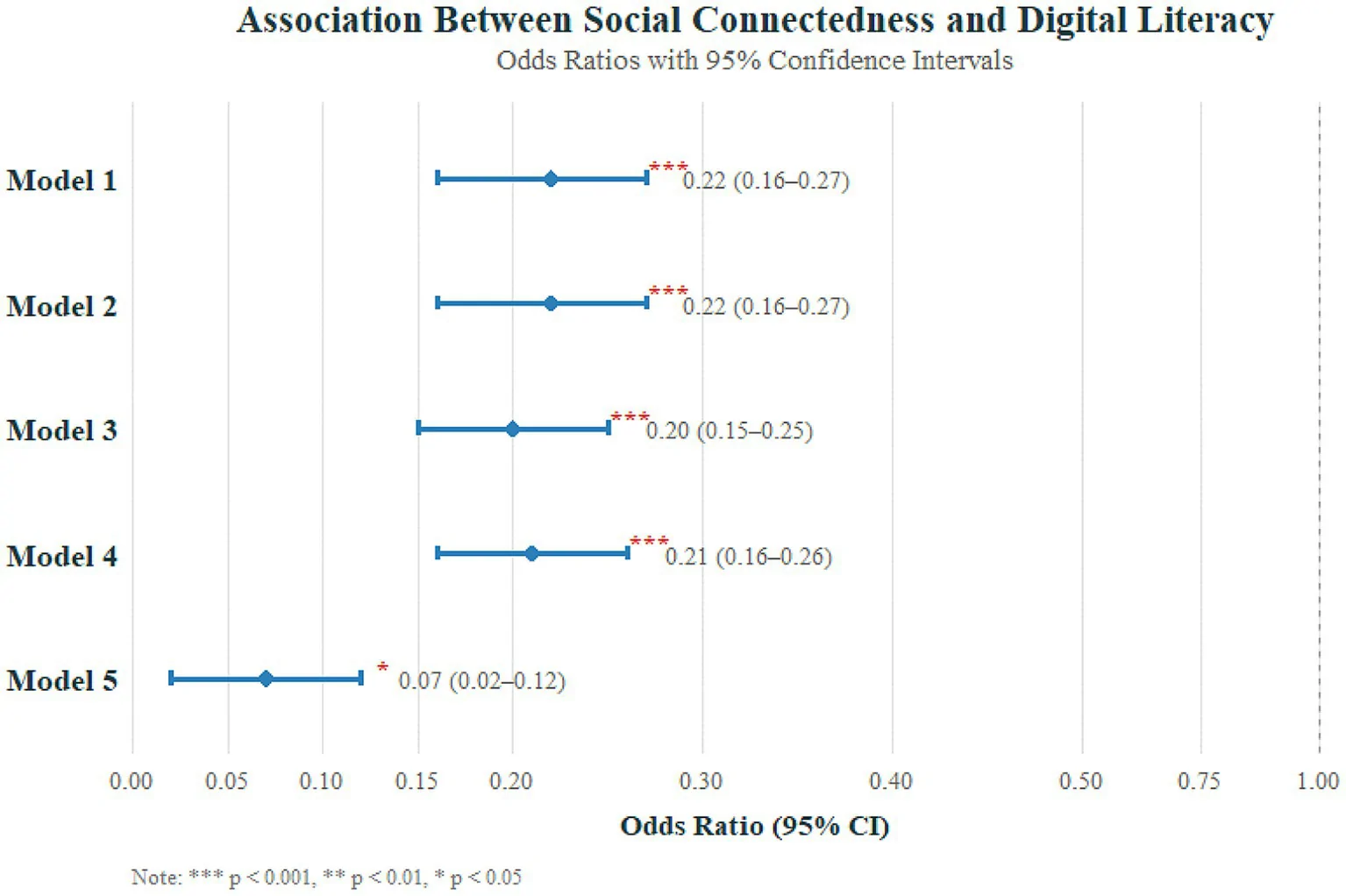
Association between social connectedness and digital literacy among Chinese older adults. Model 1: Unadjusted models. Model 2: Adjusted for age and gender. Model 3: Adjusted for age, gender, education, and retirement status. Model 4: Adjusted for age, gender, education, retirement status, income, grandchildren, and household size. Model 5: Adjusted for age, gender, education, retirement status, income, grandchildren, household size, response mode, perceived barriers, and social integration. SC, social connectedness; DL, digital literacy.

As presented in Table 2 and Figure 2, five hierarchical regression models were fitted to examine the relationship between social connectedness and digital literacy. In Model 1 (unadjusted), social connectedness was significantly associated with digital literacy (*β* = 0.22, 95% CI [0.16–0.27], *p* < 0.001). After adjusting for age and gender (Model 2), the coefficient remained stable (*β* = 0.22, *p* < 0.001). When further adjusting for education and retirement (Model 3) and additional covariates including income, grandchildren, and household size (Model 4), the coefficient remained significant but slightly attenuated (*β* = 0.20–0.21, *p* < 0.001). In the full Model 5, after adding perceived barriers (PB) and social integration (SI), the coefficient for social connectedness decreased substantially and remained statistically significant, though substantially attenuated (*β* = 0.07, 95% CI [0.02–0.12], *p* < 0.05). Both PB (*β* = −0.17, 95% CI [−0.22 to −0.12], *p* < 0.001) and SI (*β* = 0.38, 95% CI [0.32–0.44], *p* < 0.001) were significantly associated with digital literacy, supporting their roles as mediators.

**Table 2:** Hierarchical linear regression models predicting digital literacy (DL) by social connectedness (SC).

Model	Variable	95% CI	Statistic	*p*-value
Model 1	SC	0.22 (0.16–0.27)	7.98	<0.001
Model 2	SC	0.22 (0.16–0.27)	8.06	<0.001
	Age (61–70 years)	−0.19 (−0.32–−0.07)	−3.04	<0.01
	Gender (Male)	0.13 (0.04–0.22)	2.93	<0.01
Model 3	SC	0.20 (0.15–0.25)	7.49	<0.001
	Age (61–70 years)	−0.17 (−0.31–−0.03)	−2.39	<0.05
	Gender (Male)	0.11 (0.02–0.20)	2.43	<0.05
	Education (High school)	0.15 (−0.02–0.31)	1.77	0.077
	Education (College or above)	0.32 (0.17–0.47)	4.24	<0.001
	Retirement (Retired)	−0.01 (−0.11–0.10)	−0.12	0.906
Model 4	SC	0.21 (0.16–0.26)	7.7	<0.001
	Age (61–70 years)	−0.17 (−0.31–0.03)	−2.34	<0.05
	Gender (Male)	0.12 (0.02–0.21)	2.47	<0.05
	Education (High school)	0.13 (−0.04–0.29)	1.5	0.135
	Education (College or above)	0.27 (0.10–0.43)	3.24	<0.01
	Retirement (Retired)	−0.04 (−0.15–0.07)	−0.7	0.482
	Insurance (Middle income)	0.01 (−0.09–0.12)	0.22	0.824
	Insurance (High income)	0.12 (−0.02–0.26)	1.66	0.097
	Grandchildren (1)	−0.02 (−0.14–0.09)	−0.37	0.712
	Grandchildren (≥2)	−0.11 (−0.23–0.01)	−1.83	0.069
	Household size (3 persons)	0.03 (−0.09–0.14)	0.48	0.63
	Household size (≥4 persons)	−0.06 (−0.16–0.05)	−1.04	0.301
Model 5	SC	0.07 (0.02–0.12)	2.50	<0.05
	Age (61–70 years)	−0.03 (−0.15–0.09)	−0.50	0.617
	Gender (Male)	0.09 (0.02–0.16)	2.15	<0.05
	response mode (Self-administered vs. Assisted)	0.15 (0.03–0.27)	2.20	<0.05
	Education (High school)	0.12 (−0.02–0.26)	1.68	0.093
	Education (College or above)	0.26 (0.12–0.40)	3.60	<0.001
	Retirement (Retired)	−0.01 (−0.10–0.08)	−0.30	0.764
	Insurance (Middle income)	−0.02 (−0.11–0.07)	−0.50	0.617
	Insurance (High income)	0.11 (−0.01–0.23)	1.70	0.089
	Grandchildren (1)	−0.04 (−0.14–0.06)	−0.80	0.424
	Grandchildren (≥2)	−0.12 (−0.22 – −0.02)	−2.30	<0.05
	Household size (3 persons)	0.01 (−0.08–0.10)	0.20	0.842
	Household size (≥4 persons)	−0.08 (−0.17–0.01)	−1.80	0.072
	PB	−0.17 (−0.22 – −0.12)	−6.50	<0.001
	SI	0.38 (0.32–0.44)	12.00	<0.001

Model 1: unadjusted models. Model 2: adjusted for age and gender.

Model 3: adjusted for age, gender, education, and retirement status.

Model 4: adjusted for age, gender, education, retirement status, income, grandchildren, and household size.

Model 5: adjusted for age, gender, education, retirement status, income, grandchildren, household size, response mode, perceived barriers, and social integration.

SC, social connectedness; PB, perceived barriers; SI, social integration; DL, digital literacy.

### 3.4 Mediation analysis

Mediation analyses were performed using bootstrap resampling. Table 3 presents the mediation effects of perceived barriers and social integration. For perceived barriers, the indirect effect was significant across all models (indirect effect range: 0.0305–0.0381, all 95% CIs excluding zero). In the fully adjusted model (Model 4), the indirect effect was 0.0305 (95% CI [0.0120–0.0520]), accounting for 14.6% of the total effect. For social integration, the indirect effect was substantially larger (indirect effect range: 0.1215–0.1287), with the fully adjusted model showing an indirect effect of 0.1265 (95% CI [0.0940–0.1600]), accounting for 60.6% of the total effect. These results indicate that both perceived barriers and social integration serve as significant mediators in the relationship between social connectedness and digital literacy, with social integration playing a considerably stronger mediating role.

**Table 3:** Mediating roles of perceived barriers and social integration between social connectedness and digital literacy among Chinese older adults.

Variables	Model	Indirect effect	95% CI	Total effect	Prop_mediated	Prop. mediated_p
PB	Model1	0.03809	0.017, 0.0648	0.21872	17.42%	<0.01
	Model2	0.03673	0.01666, 0.06149	0.21749	16.89%	<0.01
	Model3	0.03279	0.01516, 0.05563	0.21040	15.58%	<0.01
	Model4	0.0305	0.0120, 0.0520	0.2088	14.6%	<0.01
SI	Model1	0.12670	0.092, 0.166	0.21872	57.93%	<0.01
	Model2	0.12151	0.09064, 0.15393	0.21749	55.87%	<0.01
	Model3	0.12874	0.09341, 0.16401	0.21040	61.19%	<0.01
	Model4	0.1265	0.0940, 0.1600	0.2088	60.6%	<0.01

Model 1: unadjusted models.

Model 2: adjusted for age and gender.

Model 3: adjusted for age, gender, education, and retirement status.

Model 4: adjusted for age, gender, education, retirement status, income, grandchildren, and household size, response mode.

SC, social connectedness; PB, perceived barriers; SI, social integration; DL, digital literacy.

**Figure 3:**
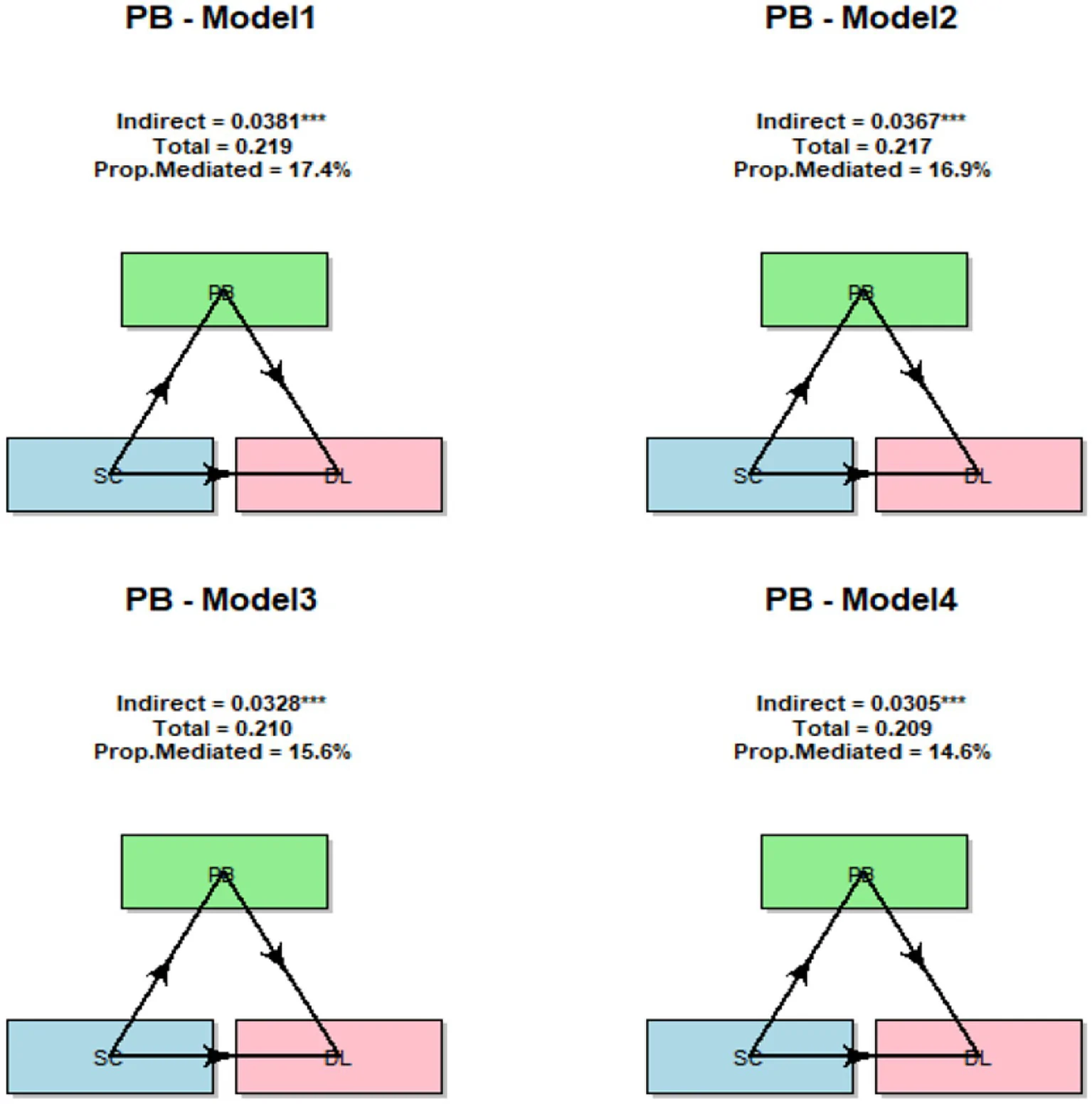
Mediation effects of perceived barriers between social connectedness and digital literacy among Chinese older adults. Model 1: Unadjusted models. Model 2: Adjusted for age and gender. Model 3: Adjusted for age, gender, education, and retirement status. Model 4: Adjusted for age, gender, education, retirement status, income, grandchildren, and household size, response mode. SC, social connectedness; PB, perceived barriers; DL, digital literacy.

**Figure 4:**
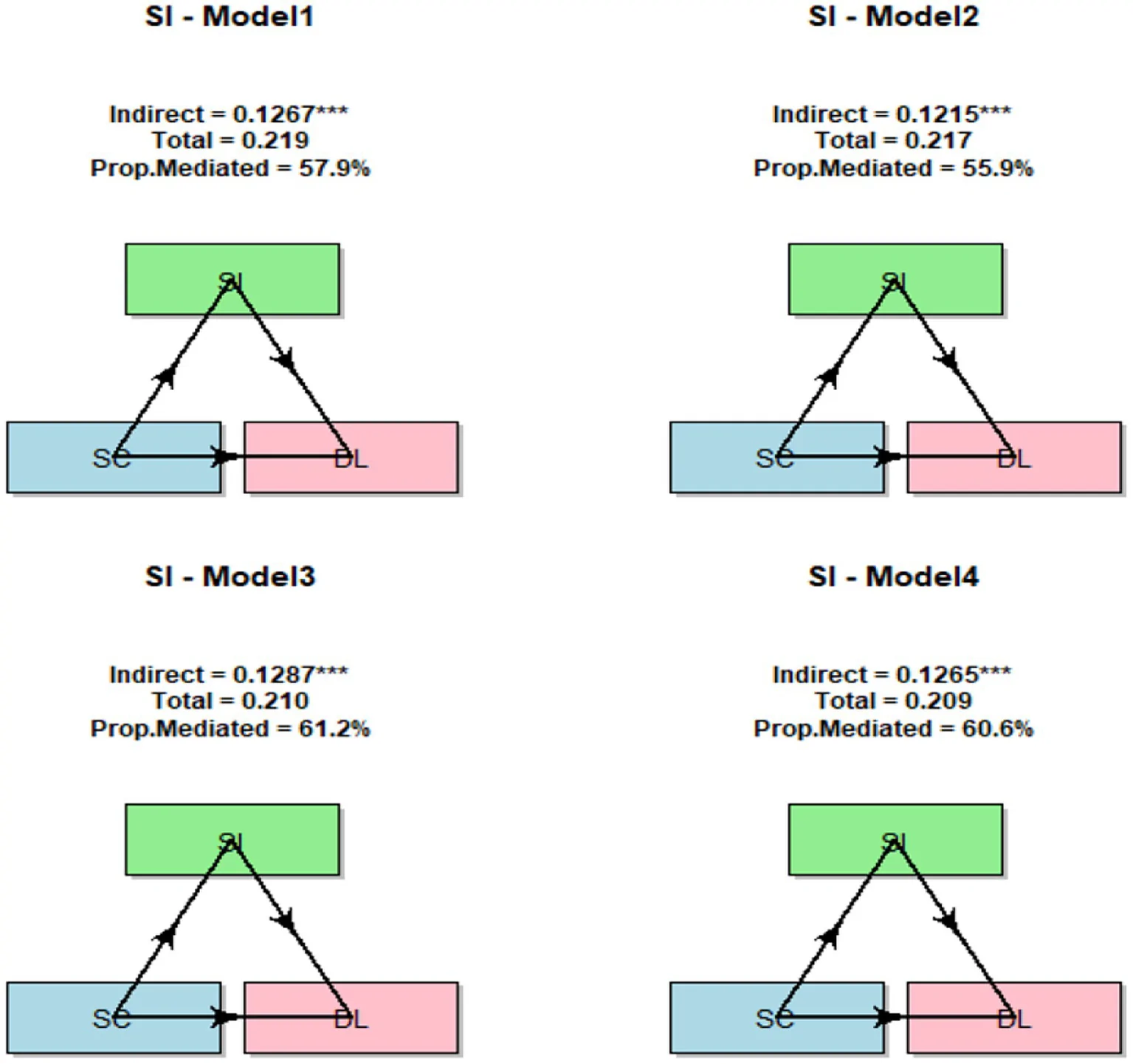
Mediation effects of social integration between social connectedness and digital literacy among Chinese older adults. Model 1: Unadjusted models. Model 2: Adjusted for age and gender. Model 3: Adjusted for age, gender, education, and retirement status. Model 4: Adjusted for age, gender, education, retirement status, income, grandchildren, and household size, response mode. SC, social connectedness; SI, social integration; DL, digital literacy.

As illustrated in Figures 3, 4, the mediation model shows that social connectedness is associated with digital literacy through two separate pathways: a negative pathway via perceived barriers (indirect effect: 0.0305, 95% CI [0.0120–0.0520]) and a positive pathway via social integration (indirect effect: 0.1265, 95% CI [0.0940–0.1600]). All path coefficients were statistically significant at *p* < 0.05. Importantly, these indirect effects should be interpreted as statistical associations consistent with mediation, not as causal evidence.

## 4 Discussion

This study examined the mediating roles of perceived barriers and social integration in the relationship between social connectedness and digital literacy among Chinese older adults. Our findings provide novel evidence that social connectedness is associated with digital literacy through two separate pathways: a negative pathway via heightened perceived barriers and a positive pathway via diminished social integration. Importantly, the mediation effect of social integration (60.6%) was substantially larger than that of perceived barriers (14.6%), highlighting the critical role of social connectedness in relation to digital inclusion in later life. These findings advance theoretical understanding of the digital divide and suggest potential directions for intervention design.

Our regression analyses revealed a significant positive association between social connectedness and digital literacy (*β* = 0.22, 95% CI [0.16–0.27], *p* < 0.001), indicating that older adults with stronger social networks tended to have higher digital literacy. This finding aligns with a growing body of literature documenting the relationship between social networks and digital competence in later life. A large-scale cross-sectional study of 9,297 Korean older adults (14) found that lower levels of offline social participation were associated with poorer digital literacy, with engaging in more than two types of social activities significantly increasing the likelihood of higher digital literacy (adjusted OR = 1.965 for two types, and 2.558 for more than three types). Similarly, a national longitudinal study using data from the China Family Panel Studies (CFPS) found that the digital divide appears to function as a double-edged sword for older adults’ mental health, with a beneficial direct pathway offset by a detrimental indirect pathway wherein motivational access undermines social capital, further supporting that digital engagement is closely intertwined with social and health-related outcomes in later life (15). A qualitative study examining older adults who were occasional or non-users of digital technology prior to the COVID-19 pandemic found that lower social connectedness and digital exclusion created a “triple exclusion” phenomenon, wherein lack of digital literacy and limited social interaction mutually reinforced each other (16). These findings converge to suggest that social connectedness is not merely a consequence of poor digital literacy but may also be a key factor associated with the capacity and motivation to engage with digital technologies. A systematic review and meta-analysis of 11 studies (17) involving over 350,000 participants found that socially or digitally disconnected seniors had a 60% higher likelihood of depressive symptoms (pooled OR = 1.60, 95% CI [1.39–1.84], *p* < 0.001), suggesting the strong interconnection between social connectedness and digital exclusion. A commentary on social isolation and loneliness among older adults reported that nearly 35% of older Americans felt lonely before the COVID-19 pandemic, with the pandemic further intensifying these feelings (18). Given that nearly 35% of older adults experienced loneliness before the pandemic—a figure that has since intensified—the importance of considering social connectedness in the context of digital exclusion has become increasingly evident. A scoping review of reviews found that various ICT interventions may have predominantly positive impacts on mitigating lower social connectedness and loneliness in community-dwelling older adults, although methodological diversity and contradictory findings complicate definite conclusions, and barriers such as individual competencies, access, and intervention design remain significant obstacles (19). Moreover, a study (20) characterizing different patterns of digital competence among 315 older Korean adults found that individuals with unskilled, passive participation, or low activity profiles had higher levels of loneliness and social isolation compared to those with active digital participation, further supporting the view that social connectedness and digital literacy are tightly intertwined.

Our mediation analyses revealed that perceived barriers significantly mediated the relationship between social connectedness and digital literacy (indirect effect: 0.0305, 95% CI [0.0120–0.0520], accounting for 14.6% of the total effect). This finding suggests that older adults with lower social connectedness tend to have less technical guidance and social support from family, friends, or community networks, making them more likely to perceive digital operations as “too difficult” or “impossible to learn,” which are in turn associated with lower digital literacy. A recent cross-sectional study (21) among 1,703 older adults in Vietnam found that digital exclusion was significantly associated with lower levels of social connectedness, suggesting that the relationship between digital non-use and social disconnectedness may be associated with unmeasured intermediate factors such as lack of social support, technological anxiety, or low self-efficacy.

The Technology Acceptance Model (TAM) provides a useful theoretical lens for interpreting this pathway. TAM posits that perceived ease of use is a core determinant of technology adoption (22). For older adults, perceived barriers encompass age-related physical limitations, cognitive difficulties, technophobia, distrust, and lack of assistance. A rapid review (9) found that interventions may be associated with fewer barriers and higher older adults’ digital literacy and self-efficacy, with individuals at risk of lower social connectedness benefiting most. A cross-sectional study of 233 older adults in Beijing (23) demonstrated that personal mastery, a psychological resource, was significantly associated with higher digital literacy, whereas perceived constraints had minimal effect, suggesting that older adults with lower social connectedness may lack the social reinforcement that builds personal mastery, thereby exacerbating perceived barriers. Research from Mexico City using TAM (24) found that technological anxiety is associated with a critical barrier, while social influence is associated with perceived ease of use, though the quality of support matters: pressuring or impatient interactions may diminish perceived usefulness. For older adults with lower social connectedness who lack consistent, patient support networks, even limited interactions may be associated with further entrenchment of perceived barriers. A study on technology training for older adults emphasized that well-designed programs may be associated with higher levels of both independence and social connections, and that TAM’s perceived usefulness may be associated with older adults embracing digital tools (25). However, our findings suggest that perceived ease of use (freedom from perceived barriers) may be equally or more important for older adults with lower social connectedness who lack the social scaffolding that typically reduces technological anxiety. A scoping review (26) of barriers to digital education programs for older adults identified multiple levels—individual, situational, institutional, and contextual—that are associated with intervention success, aligning with our finding that perceived barriers mediate between social connectedness (social-structural) and digital literacy (individual competency). Thus, interventions may need to address both individual technophobia and situational factors such as access to patient, knowledgeable social support. Additionally, our application of the Technology Acceptance Model focused solely on perceived ease of use (operationalized as perceived barriers), as the perceived usefulness scale did not meet validity criteria. Future research should examine both TAM dimensions to capture the full technology acceptance process among older adults.

Our analyses revealed that social integration served as a stronger mediator than perceived barriers, accounting for 60.6% of the total effect (indirect effect: 0.1265, 95% CI [0.0940–0.1600]). This indicates that older adults with lower social connectedness, with smaller networks and lower interaction frequency, struggle to access digital skills information and encouragement, which is associated with lower digital literacy. One study (27) also found that social support acts as a partial mediator in the relationship between digital literacy and the health status of senior citizens, suggesting a critical role of social integration in the relationship between digital competence and tangible outcomes.

The active aging framework (health, participation, security) provides theoretical support: social participation offers observational learning, peer support, and technological demonstration essential for digital literacy (13). National longitudinal data show that digital literacy is associated with older adults’ employment participation, and this association is linked to aging attitudes, strengthening social capital, and alleviating grandchild care burdens. Our findings complement this by showing the reverse direction—from social integration to digital literacy—is equally important (28). A Korean cross-sectional study (14) found that lower social participation was associated with poorer digital literacy, with a dose–response relationship (aOR = 1.965 for two types, aOR = 2.558 for three+ types); however, senior community activities were negatively associated (aOR = 0.762), indicating not all participation is equally beneficial. Research on socialization agents identified three processes—reciprocity, self-socialization, and outsourcing—through which older adults learn digital skills; individuals with lower social connectedness lack access to these processes, consistent with our finding that social integration accounts for most of the total effect. Studies on retirement migrants (29) and aging attitudes in China suggest that digital literacy is associated with social integration and is associated with fewer negative aging attitudes (loneliness, isolation), indicating a reciprocal relationship. The stronger mediation effect of social integration (60.6% vs. 14.6%) likely reflects the collectivist orientation of Chinese society, where social relationships centrally shape individual outcomes. Among the five digital literacy dimensions, higher-order competencies such as Digital Content Creation (DCC) and Digital Information Evaluation (DIE) may be particularly susceptible to lower social connectedness, as they rely more on social learning, peer modeling, and collaborative practice than basic operational skills.

It is worth noting that the two indirect effects were estimated from separate simple mediation models; thus, direct comparison of their magnitudes is descriptive and should be interpreted with caution. Nevertheless, their 95% bootstrap confidence intervals showed no overlap (SI: 0.0940–0.1600; PB: 0.0120–0.0520), suggesting that the difference is unlikely due to chance alone. Our mediation model revealed that social integration was a substantially stronger mediator than perceived barriers (indirect effect: 0.1265 vs. 0.0305; proportion of total effect: 60.6% vs. 14.6%). From a theoretical perspective, social integration directly provides observational learning, peer support, and technological demonstration—mechanisms essential for digital skill acquisition. In contrast, perceived barriers operate as an internal psychological filter (e.g., technophobia, low self-efficacy). Additionally, our sample consisted of relatively young older adults (aged 50–70 years), among whom technological anxiety and physical barriers may be less pronounced compared to the oldest old. This age profile may partly explain why the perceived-barriers pathway accounted for a smaller proportion of the total effect. A grounded theory study on digital exclusion of rural older adults patients found that family support is associated with a stronger relationship between social integration and digital literacy, which aligns with our finding that social integration plays a dominant role. Previous research (30) also indicates that digital literacy is associated with older adults’ outcomes partly by alleviating grandchild care burdens, consistent with our finding that participants with two or more grandchildren had significantly lower digital literacy. Although we tested the two pathways separately, they are not necessarily independent. Older adults with lower social connectedness and limited social support may perceive greater technological difficulties, which could further discourage their social participation, potentially forming a reinforcing cycle. Future studies should examine these possible cascading or reciprocal effects using longitudinal or cross-lagged designs. Finally, the direct effect of social connectedness remained statistically significant, though substantially attenuated, suggesting a need for investigation of additional unmeasured mechanisms (e.g., self-efficacy, health status, digital infrastructure).

Our findings suggest several potential directions for intervention, although causal inference is not warranted given the cross-sectional design. For the perceived barriers pathway, interventions that may be associated with lower technological anxiety—such as low-stress learning environments, peer mentoring, or simplified interface design—may be worth exploring. For the social integration pathway, strengthening social networks through community-based digital learning groups, intergenerational mentoring, or leveraging existing community centers as digital hubs could be considered. Older adults with lower social connectedness (e.g., those living alone or with limited family contact) may require more proactive outreach. However, these suggestions are derived from statistical associations and could be tested in future longitudinal or experimental studies before implementation.

Several limitations should be acknowledged. First, due to the cross-sectional design, causal inference is precluded. Reverse causation (e.g., higher digital literacy enabling older adults to build stronger social networks) or bidirectional relationships between social connectedness and digital literacy cannot be ruled out. Our hypothesized direction is primarily theory-driven, but unmeasured confounding (e.g., personality traits or health status) may also exist. Longitudinal studies with repeated measures are needed to formally test the temporal ordering and reciprocal effects. This represents a promising direction for future research. Second, our sample was skewed toward younger (85.3% aged 51–60) and more highly educated (63.4% with associate degree or above) older adults, compared with national estimates for this age group. Thus, findings may not be generalizable to older, less educated populations. Third, self-reported measures may introduce social desirability bias, especially for perceived barriers and digital literacy; future research should incorporate objective or informant-based assessments. Fourth, our measure of social integration focused on neighborhood interaction and community engagement, missing other forms (e.g., online networks, religious participation) that may have differential effects: as suggested by findings that senior community activities were negatively associated with digital literacy among Korean older adults. Fifth, the small sample size for certain subgroups (e.g., participants with ≥2 grandchildren, *n* = 135) limited statistical power for detecting subgroup differences. Sixth, although we examined perceived barriers and social integration as mediators, other mechanisms (e.g., self-efficacy, health status, digital infrastructure) may also play important roles; the statistically significant direct effect of social connectedness after accounting for both mediators calls for exploration of additional pathways, such as self-efficacy based on social cognitive theory. Despite these limitations, this study provides novel evidence for the dual mediation model linking social connectedness to digital literacy in later life. Seventh, we acknowledge that the 47-item digital literacy scale may have imposed a response burden on older participants, particularly for those who required assistance, potentially affecting data quality. Although we employed a mixed-mode design to accommodate varying levels of literacy and cognitive capacity, the length of the questionnaire may still have contributed to participant fatigue. Future studies should consider shorter versions or adaptive administration procedures to reduce respondent burden. Finally, no formal cognitive screening was administered; we used only an attention-check question to identify random responses. Although our mixed-mode design allowed individuals with lower comprehension to participate, those with severe cognitive or communication impairments were excluded. Thus, subclinical cognitive differences may still confound the observed associations. Future studies should include objective cognitive measures to examine the unique contribution of social connectedness to digital literacy. Additionally, our application of the Technology Acceptance Model focused solely on perceived ease of use (operationalized as perceived barriers), as the perceived usefulness scale did not meet validity criteria. Future research should examine both TAM dimensions to capture the full technology acceptance process among older adults.

Future studies should adopt longitudinal designs, employ objective performance-based measures of digital literacy, assess diverse forms of social integration across different cultural contexts, recruit larger and more heterogeneous samples, and examine additional mediating mechanisms including self-efficacy and health status.

## 5 Conclusion

This study suggests that perceived barriers and social integration mediate the relationship between social connectedness and digital literacy among Chinese older adults. Lower social connectedness is associated with lower digital literacy. Higher levels of perceived barriers and lower levels of social integration were both associated with the observed association between social connectedness and digital literacy. The mediation effect of social integration is substantially stronger than that of perceived barriers, suggesting the critical role of social connectedness. Interventions may consider a dual-pronged approach that addresses technological anxiety while strengthening social networks, particularly for older adults with lower social connectedness. However, the cross-sectional design precludes causal inference. Future research could employ longitudinal designs to validate these findings.

## Data Availability

The raw data supporting the conclusions of this article will be made available by the authors, without undue reservation.
