# Supplementary material for: Social connectedness and digital literacy in later life: the mediating roles of perceived barriers and social integration
Source: Front Public Health. 2026 Sep 9;14:1849056. doi: 10.3389/fpubh.2026.1849056 (PMC13597926; doi:10.3389/fpubh.2026.1849056)
Supplement: Supplementary file 1 [file Data_sheet_1.DOCX]

**Supplementary Material**

**Table S1 Descriptive statistics of core variables and digital literacy dimensions**

| **Statistic** | **SC** | **PB** | **SI** | **BKN** | **IAA** | **DIC** | **DIE** | **DCC** |
| --- | --- | --- | --- | --- | --- | --- | --- | --- |
| Min | 1.33 | 1 | 1.38 | 1.5 | 1 | 1 | 1 | 1 |
| P5 | 2.2 | 1.38 | 2.38 | 3.51 | 2.39 | 2.6 | 2.33 | 1.78 |
| P10 | 2.67 | 1.5 | 2.75 | 3.86 | 2.88 | 3 | 2.67 | 2.11 |
| P25 | 3.17 | 1.88 | 3.25 | 4.21 | 3.5 | 3.6 | 3.33 | 2.67 |
| Median | 3.83 | 2.5 | 3.75 | 4.5 | 4 | 4 | 3.67 | 3.33 |
| Mean | 3.75 | 2.5 | 3.64 | 4.39 | 3.87 | 3.94 | 3.65 | 3.24 |
| P75 | 4.33 | 3 | 4.25 | 4.71 | 4.38 | 4.4 | 4.33 | 4 |
| P90 | 4.83 | 3.58 | 4.38 | 4.86 | 4.6 | 4.6 | 4.53 | 4.33 |
| P95 | 5 | 3.88 | 4.5 | 4.93 | 4.75 | 4.8 | 4.67 | 4.44 |
| Max | 6 | 5 | 5 | 5 | 5 | 5 | 5 | 5 |

Abbreviations: SC = social connectedness; PB = perceived barriers; SI = social integration; IAA = Information Acquisition and Application; BKN = Basic Knowledge and Operation; DCC = Digital Content Creation; DIC = Digital Information Communication; DIE = Digital Information

Figure S1. Correlation Matrix of DL Variables

Abbreviations: IAA = Information Acquisition and Application; BKN = Basic Knowledge and Operation; DCC = Digital Content Creation; DIC = Digital Information Communication; DIE = Digital Information.

**Table S2. Variable coding and types used in descriptive statistics, regression and mediation models**

| **Variable** | **Type in models** | **Coding / Categories** |
| --- | --- | --- |
| SC | Continuous | Range 0–6 (higher = stronger network) |
| PB | Continuous | Range 1–5 (higher = greater barriers) |
| SI | Continuous | Range 1–5 (higher = greater integration) |
| DL | Continuous | Range 1–5 (higher = better literacy) |
| BKN | Continuous | Range 1–5 |
| IAA | Continuous | Range 1–5 |
| DIC | Continuous | Range 1–5 |
| DIE | Continuous | Range 1–5 |
| DCC | Continuous | Range 1–5 |
| age | Categorical (dummy) | 1 = 51–60 years, 2 = 61–70 years |
| gender | Categorical (dummy) | 0 = female, 1 = male |
| education | Categorical (dummy) | 1 = junior high or below, 2 = high school/technical, 3 = associate degree or above |
| insurance | Categorical (dummy) | 1 = low (≤4999 RMB), 2 = middle (5000–9999 RMB), 3 = high (≥10000 RMB) |
| retirement | Categorical (dummy) | 0 = not retired, 1 = retired |
| grandchildren | Categorical (dummy) | 1 = none, 2 = one, 3 = two or more |

Abbreviations: SC = social connectedness; PB = perceived barriers; SI = social integration; DL = digital literacy; BKN = basic knowledge and norms; IAA = information acquisition and application; DIC = digital interaction and communication; DIE = digital information evaluation; DCC = digital content creation.

Note: All continuous variables were checked for normality and used as continuous in linear regression and mediation models. Tertile groupings (low/moderate/high) for SC, PB, and SI were created only for descriptive comparisons (one‑way ANOVA and Figure 1) and were not used in inferential models.

**Table S3. Model fit indices for the confirmatory factor analysis of the digital literacy scale (N = 505)**

| **Fit Index** | **Value** | **Threshold** | **Evaluation** |
| --- | --- | --- | --- |
| χ²/df | 1.66 | < 3.00 | Good |
| CFI (Robust) | 0.952 | ≥ 0.90 | Acceptable to good |
| TLI (Robust) | 0.947 | ≥ 0.90 | Acceptable to good |
| RMSEA (Robust) | 0.036 | ≤ 0.08 | Good |
| 90% CI for RMSEA | [0.032, 0.041] | — | — |
| SRMR | 0.042 | ≤ 0.08 | Good |

Note: MLR (maximum likelihood estimation with robust standard errors) estimator was used to account for non-normality. CFI = comparative fit index; TLI = Tucker–Lewis index; RMSEA = root mean square error of approximation; SRMR = standardized root mean square residual.
